# 1990–2021 global, regional, and national analysis of the burden and trends of non-alcoholic fatty liver disease

**DOI:** 10.3389/fmed.2025.1609816

**Published:** 2025-06-06

**Authors:** Jun Tang, Nan Zheng, Yu-Xin Yan, Nan Zhang, Xiao-Mei Ren

**Affiliations:** School of Public Health, Shaanxi University of Chinese Medicine, Xianyang, China

**Keywords:** non-alcoholic fatty liver disease, prevalence, incidence, mortality, disability-adjusted life years, socio-demographic index, epidemiological trends, future projections

## Abstract

**Background:**

NAFLD, a leading global cause of liver disease, is projected to dominate end-stage liver disease burden. Strongly linked to obesity, type 2 diabetes, and metabolic syndrome, its prevalence and incidence are rising rapidly, yet it remains unclassified by WHO as a priority non-communicable disease. This study aims to assess the global, regional, and national burden of NAFLD from 1990 to 2021 and provide evidence for future prevention and control strategies.

**Methods:**

Using 2021 Global Burden of Disease (GBD) data, this study analyzed NAFLD prevalence, incidence, mortality, and disability-adjusted life years (DALYs) across 204 countries (1990–2021), stratified by sex, age, and Socio-demographic Index (SDI). Trends were assessed via age-standardized rates (ASR) and estimated annual percentage changes (EAPC), with projections to 2050.

**Results:**

In 2021, global NAFLD cases surged to 1.268 billion (124.63% increase since 1990), while ASR rose from 12,085.09 to 15,018.07 per 100,000. Middle- and high-SDI regions exhibited steepest rises in incidence and mortality. Oceania, Central and Latin America had highest prevalence; North Africa and the Middle East reported peak incidence. Projections indicate escalating global incidence through 2050, disproportionately affecting females, driven by population growth and aging.

**Conclusion:**

The escalating burden underscores urgent need for region-specific interventions, particularly in aging and high-growth populations. Enhanced screening and early intervention are critical to mitigate NAFLD’s expanding impact. This study highlights actionable data to inform global public health strategies, emphasizing tailored prevention and control measures to address evolving epidemiological trends.

## 1 Introduction

Non-alcoholic fatty liver disease (NAFLD) is a chronic metabolic disorder characterized by the accumulation of fat in the liver, which is unrelated to alcohol consumption or other identifiable causes of liver damage. It is associated with various metabolic comorbidities, including obesity, type 2 diabetes, dyslipidemia, and metabolic syndrome (1). NAFLD can progress to more severe liver conditions such as non-alcoholic steatohepatitis (NASH), advanced fibrosis, cirrhosis, and hepatocellular carcinoma (2). In recent years, the incidence and prevalence of NAFLD have increased significantly worldwide, with a global prevalence of approximately 25% (3). It has surpassed viral hepatitis as the most common chronic liver disease globally (4). The prevalence of NAFLD with advanced fibrosis is increasing, and patients with progressive NAFLD have higher liver-related and non-liver-related mortality rates compared to the general population (2). However, public awareness of NAFLD remains low, and it is not recognized by the World Health Organization as a significant non-communicable disease.

Previous studies have used Global Burden of Disease (GBD) data to explore the global epidemiology of NAFLD. GBD 2019 provided the latest assessment of the global, regional, and national burden of NAFLD from 1990 to 2019, showing that the age-standardized prevalence and liver-related mortality rates of NAFLD increased globally, with worsening trends observed in 202 countries and 167 countries (5). GBD 2021 provides updated data on the incidence, prevalence, mortality, and disability-adjusted life years (DALYs) of NAFLD. In addition to informing the latest prevention and treatment measures, estimating the future burden of the disease is particularly important. Although epidemiological reports have predicted NAFLD in 7 to 8 regions (6), there has been no broader global prediction to estimate the future burden of this disease.

To address this knowledge gap, we aim to provide an updated assessment of the trends in NAFLD prevalence, incidence, mortality, and DALY rates at the global, regional, and national levels from 1990 to 2021, offering targeted prevention and treatment strategies for NAFLD.

## 2 Methods

### 2.1 Data sources

The GBD 2021 study utilizes the latest epidemiological data and refined standardized methods to comprehensively assess health losses associated with 369 diseases, injuries, and impairments across 204 countries and territories, along with 88 risk factors. Detailed information on the study design and methods of the GBD research has been widely described in existing GBD literature (7). In this study, we searched for “NAFLD” in the disease classification section, selected the age group “15 years and older,” and downloaded data for the period “1990–2021” for analysis.

### 2.2 Disease classification

According to the International Classification of Diseases, 11th Revision (ICD-11), the code for non-alcoholic fatty liver disease is DB92 (8).

### 2.3 Socio-demographic index (SDI)

The SDI quantifies the development level of a country or region based on fertility rates, education levels, and per capita income. Ranging from 0 to 1, a higher SDI indicates a higher level of socio-economic development (9). In this study, countries and regions were divided into five quintiles (low, low-middle, middle, high-middle, and high SDI regions) (10) to examine the relationship between NAFLD burden and socio-economic development.

### 2.4 Statistical methods

Data were organized using Excel, and charts were created using GraphPad Prism 9.5 software. Based on the GBD 2021 standard population, the R language was used to apply the direct standardization method to calculate age-standardized rates (ASRs) for incidence, prevalence, mortality, and DALY rates. The change rates and estimated annual percentage changes (EAPCs) were calculated to analyze the burden of NAFLD. The ASR was calculated as follows:


$$\begin{array}{c} \text{ASR}=\frac{\sum (\text{Standard PopulationComposition}\times \text{Age}-\text{SpecificRate})}{\sum \text{Standard PopulationComposition}\;}\\ \times 100,100/100,100 \end{array}$$

The EAPC is a common indicator in epidemiological studies used to determine the temporal evolution of ASRs. It is represented by the coefficient *β* derived from the natural logarithm of the ASR. Here, Y represents ln(ASR), X corresponds to the calendar year, and *ε* is the error term. The EAPC and its 95% confidence interval (CI) were determined using the following linear regression model:


Y=α+βX+ε

$$\text{EAPC}=100^{*}(\exp(\beta )-1)$$

According to this formula, a positive or negative *β* indicates an increasing or decreasing trend in ASR, respectively. If both the EAPC and the lower limit of its 95% CI are positive, the ASR is considered to have an increasing trend. Conversely, if both the EAPC and the upper limit of its 95% CI are negative, the ASR is considered to have a decreasing trend. If neither condition is met, the ASR is considered stable.

## 3 Results

### 3.1 Global level

In 2021, the global number of NAFLD cases reached 1.268 billion, representing a 124.63% increase from 1990. The age-standardized prevalence rate (ASPR) rose from 12,085.09 per 100,000 (95% UI: 11,058.41 ~ 13,184.29) in 1990 to 15,018.07 per 100,000 (95% UI: 13,756.47 ~ 16,361.44) in 2021, with an EAPC of 0.73 (95% CI: 0.67 ~ 0.79). In 2021, the global number of NAFLD incident cases reached 48,353,272.35 (95% UI: 44,229,138.51 ~ 52,358,017.44), an increase of 94.53% compared to 1990. The age-standardized incidence rate (ASIR) increased from 475.54 per 100,000 (95% UI: 432.59 ~ 518.19) in 1990 to 593.28 per 100,000 (95% UI: 542.72 ~ 643.70) in 2021, with an EAPC of 0.73 (95% CI: 0.67 ~ 0.77), indicating a continuous rise in NAFLD incidence. The number of deaths attributable to NAFLD increased significantly from 59,536.42 (95% UI: 76,300.72 ~ 45,666.08) in 1990 to 138,328.27 (95% UI: 173,904.68–108,287.75) in 2021. In 2021, the age-standardized death rate (ASDR) was 1.62 per 100,000 (95% UI: 1.27 ~ 2.02), with an EAPC of 0.19 (95% CI: 0.15 ~ 0.24). The global DALYs due to NAFLD in 2021 were 3,667,267.15 (95% UI: 2,903,575.82 ~ 4,607,306.74), an increase of 117.09% compared to 1990. The age-standardized DALY rate was 42.40 per 100,000 (95% UI: 33.60 ~ 53.31), with an EAPC of 0.16 (95% CI: 0.10 ~ 0.23) (Table 1).

**Table 1:** prevalence, incidence rate, mortality and DALY rate of NAFLD between 1990 and 2021 at the global and regional level.

Location	1990		2021		EAPC_95%CI
	Number (95%UI)	ASR (95%UI)	Number (95%UI)	ASR (95%UI)	
Prevalence					
Global	564432127.81 (516524818.33 ~ 618101250.19)	12085.09 (11058.41 ~ 13184.29)	1267867997.48 (1157934071.29 ~ 1380435423.31)	15018.07 (13756.47 ~ 16361.44)	0.73(0.67 ~ 0.79)
High SDI	86299846.18 (79056144.47 ~ 94042895.06)	8520.08 (7809.16 ~ 9274.20)	168913422.00 (154930231.62 ~ 182979398.12)	11543.68 (10585.57 ~ 12529.08)	1.09(1.05 ~ 1.14)
High-middle SDI	131193560.63 (120116956.29 ~ 143511510.02)	12230.41 (11204.06 ~ 13334.14)	260953818.21 (239296771.67 ~ 283534843.34)	15471.06 (14155.43 ~ 16878.90)	0.78(0.67 ~ 0.88)
Middle SDI	192822301.98 (176670360.94 ~ 212169963.39)	13847.03 (12679.85 ~ 15124.73)	453616039.65 (414597794.01 ~ 494648032.73)	16589.92 (15168.29 ~ 18069.08)	0.61(0.54 ~ 0.68)
Low-middle SDI	113447134.62 (103912268.16 ~ 124978282.10)	13578.98 (12385.81 ~ 14820.28)	275895435.34 (251999522.26 ~ 301392883.10)	15748.70 (14418.61 ~ 17126.74)	0.49(0.44 ~ 0.53)
Low SDI	40088807.25 (36552295.15 ~ 44255673.04)	12421.48 (11351.33 ~ 13593.54)	107435458.50 (98307358.33 ~ 118879398.80)	13889.22 (12685.44 ~ 15166.26)	0.35(0.32 ~ 0.39)
Incidence					
Global	24856158.66 (22579697.49 ~ 27333109.87)	475.54 (432.59 ~ 518.19)	48353272.35 (44229138.51 ~ 52358017.44)	593.28 (542.72 ~ 643.70)	0.73(0.69 ~ 0.77)
High SDI	3262720.60 (2977823.55 ~ 3550597.02)	342.72 (312.23 ~ 373.46)	5326140.81 (4910092.39 ~ 5729423.93)	450.03 (412.20 ~ 488.43)	1.00(0.95 ~ 1.05)
High-middle SDI	5404817.63 (4893944.23 ~ 5906253.77)	478.36 (435.28 ~ 522.10)	8509328.08 (7857781.78 ~ 9203998.94)	611.29 (557.97 ~ 665.56)	0.80(0.71 ~ 0.89)
Middle SDI	9002611.37 (8141914.15 ~ 9945635.92)	533.69 (485.35 ~ 580.65)	17017312.52 (15552700.12 ~ 18416002.69)	656.97 (602.08 ~ 712.86)	0.68(0.65 ~ 0.72)
Low-middle SDI	5235751.86 (4734019.63 ~ 5777133.43)	520.74 (474.21 ~ 566.64)	12064461.85 (10980813.99 ~ 13233265.32)	623.22 (569.61 ~ 676.22)	0.59(0.55 ~ 0.62)
Low SDI	1925958.28 (1735020.47 ~ 2127906.34)	483.42 (440.22 ~ 527.63)	5396909.32 (4857493.86 ~ 5987672.28)	553.74 (503.53 ~ 605.08)	0.44(0.41 ~ 0.47)
Deaths					
Global	59536.42 (76300.72 ~ 45666.08)	1.53 (1.17 ~ 1.97)	138328.27 (173904.68 ~ 108287.75)	1.62 (1.27 ~ 2.02)	0.19(0.15 ~ 0.24)
High SDI	15519.21 (20040.59 ~ 11783.90)	1.43 (1.09 ~ 1.84)	30012.28 (37480.17 ~ 23144.76)	1.48 (1.15 ~ 1.85)	0.13(0.05 ~ 0.21)
High-middle SDI	14182.33 (18099.03 ~ 10777.34)	1.47 (1.12 ~ 1.86)	27009.07 (34199.42 ~ 20896.72)	1.4 (1.09 ~ 1.76)	−0.14(−0.29 ~ 0.00)
Middle SDI	16083.30 (20436.77 ~ 12373.16)	1.63 (1.25 ~ 2.11)	46547.06 (58521.71 ~ 36615.28)	1.78 (1.39 ~ 2.21)	0.36(0.33 ~ 0.40)
Low-middle SDI	9647.71 (13467.44 ~ 6956.52)	1.69 (1.20 ~ 2.43)	26193.36 (33981.28 ~ 19436.72)	1.87 (1.39 ~ 2.41)	0.37(0.35 ~ 0.39)
Low SDI	4026.18 (5554.95 ~ 2906.33)	1.89 (1.0.34 ~ 2.67)	8414.82 (10874.84 ~ 6555.18)	1.74 (1.34 ~ 2.23)	−0.32 (−0.36 ~ −0.28)
DALY					
Global	1689254.11 (1292477.3 ~ 2206534.47)	40.20 (30.73 ~ 52.23)	3667267.15 (2903575.82 ~ 4607306.74)	42.40 (33.60 ~ 53.31)	0.16(0.10 ~ 0.23)
High SDI	404317.25 (313336.30 ~ 522662.26)	38.39 (29.45 ~ 49.78)	697587.39 (548692.55 ~ 876315.94)	38.73 (30.51 ~ 48.83)	0.02(−0.07 ~ 0.11)
High-middle SDI	386000.16 (294436.27 ~ 496602.78)	37.79 (28.89 ~ 48.32)	713364.87 (554265.67 ~ 916764.94)	38.00 (29.71 ~ 48.17)	0.02(−0.19 ~ 0.23)
Middle SDI	491313.01 (375149.19 ~ 633402.85)	42.14 (32.23 ~ 54.23)	1260023.95 (991413.95 ~ 1590486.71)	45.53 (36.02 ~ 56.74)	0.25(0.22 ~ 0.29)
Low-middle SDI	284746.57 (207623.14 ~ 401333.50)	41.94 (30.32 ~ 58.47)	740423.77 (563620.12 ~ 963481.73)	47.65 (35.77 ~ 61.75)	0.46(0.43 ~ 0.49)
Low SDI	120721.30 (88326.47 ~ 164481.86)	47.45 (34.47 ~ 64.59)	251891.42 (195512.17 ~ 324225.52)	42.89 (33.61 ~ 55.57)	−0.42(−0.46 ~ −0.38)

### 3.2 Regional level

In 2021, the global burden of NAFLD showed significant regional variations, closely related to SDI levels. The middle SDI region had the highest ASPR and ASIR, at 16,589.92 per 100,000 (95% UI: 15,168.29 ~ 18,069.08) and 656.97 per 100,000 (95% UI: 602.08 ~ 712.86), respectively, while the high SDI region had the lowest ASPR and ASIR, at 11,543.68 per 100,000 (95% UI: 10,585.57 ~ 12,529.08) and 450.03 per 100,000 (95% UI: 412.20 ~ 488.43), respectively. The high-middle SDI region experienced the largest increase in ASPR and ASIR, with ASPR rising from 12,230.41 per 100,000 (95% UI: 11,204.06 ~ 13,334.14) in 1990 to 15,471.06 per 100,000 (95% UI: 14,155.43 ~ 16,878.90) in 2021, with an EAPC of 0.78 (95% CI: 0.67 ~ 0.88), and ASIR increasing from 478.36 per 100,000 (95% UI: 435.28 ~ 522.10) in 1990 to 611.29 per 100,000 (95% UI: 557.97 ~ 665.56) in 2021, with an EAPC of 0.80 (95% CI: 0.71 ~ 0.89). In contrast, the low SDI region had the smallest increase in ASPR and ASIR, with ASPR rising from 12,421.48 per 100,000 (95% UI: 11,351.33 ~ 13,593.54) in 1990 to 13,889.22 per 100,000 (95% UI: 12,685.44 ~ 15,166.26) in 2021, with an EAPC of 0.35 (95% CI: 0.32 ~ 0.39), and ASIR increasing from 483.42 per 100,000 (95% UI: 440.22 ~ 527.63) in 1990 to 553.74 per 100,000 (95% UI: 503.53 ~ 605.08) in 2021, with an EAPC of 0.44 (95% CI: 0.41 ~ 0.47). The ASDR further highlighted these regional differences, with the low-middle SDI region having the highest ASDR and the high-middle SDI region having the lowest. The ASDR in the low-middle SDI region was 1.87 per 100,000 (95% UI: 1.39 ~ 2.41), while in the high-middle SDI region, it was 1.40 per 100,000 (95% UI: 1.09 ~ 1.76). The disease burden quantified by age-standardized DALY rates further emphasized these regional disparities. The low-middle SDI region had the highest age-standardized DALY rate at 47.65 per 100,000 (95% UI: 35.77 ~ 61.75), while the high-middle SDI region had the lowest at 38.00 per 100,000 (95% UI: 29.71 ~ 48.17) (Table 1).

### 3.3 National level

**Figure 1:**
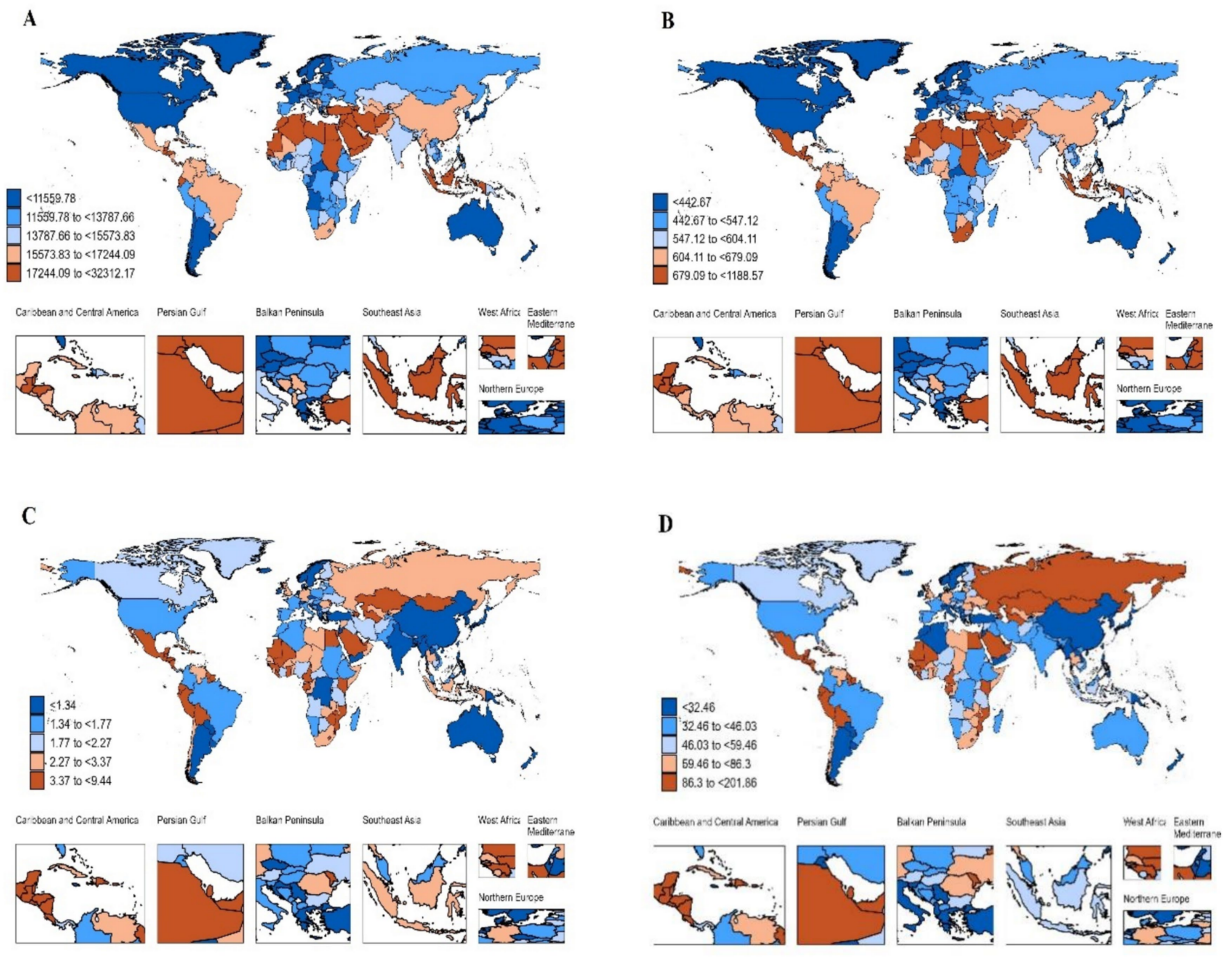
The global disease burden of NAFLD for both sexes in 204 countries and territories. **(A)** Prevalence rate; **(B)** Incidence rate; **(C)** death rate; **(D)** DALY rate.

The study results indicate that Oceania bears the highest global burden of NAFLD prevalence, with the highest ASPR at 27,686.69 per 100,000 (95% UI: 25,586.92 ~ 29,914.62), followed by Central America at 16,983.98 per 100,000 (95% UI: 15,536.52 ~ 18,533.59). Latin America also showed a high ASPR at 16,662.75 per 100,000 (95% UI: 15,244.95 ~ 18,205.46) (Table 1; Figure 1A). The incidence of NAFLD in North Africa and the Middle East is also of significant concern, with these regions having the highest global ASIR at 1,037.64 per 100,000 (95% UI: 963.01 ~ 1,109.65). In contrast, the high-income Asia-Pacific region had the lowest ASIR at 348.79 per 100,000 (95% UI: 319.19 ~ 378.61) (Table 1; Figure 1B). Additionally, by analyzing the ASDR and age-standardized DALY rates due to NAFLD in 2021, we observed consistency among the most affected countries. Latin America and Central America had significantly higher ASDR and age-standardized DALY rates, with the highest ASDR at 5.89 per 100,000 (95% UI: 4.03 ~ 8.07) and 5.00 per 100,000 (95% UI: 3.74 ~ 6.45), respectively, and the highest age-standardized DALY rates at 142.16 per 100,000 (95% UI: 98.6 ~ 198.78) and 138.84 per 100,000 (95% UI: 104.37 ~ 180.55), respectively. Eastern Europe had the largest increase in NAFLD ASDR (EAPC 3.58, 95% CI: 2.95 ~ 4.22), while the high-income Asia-Pacific region had the largest decrease in ASDR (EAPC -2.09, 95% CI: −2.24 ~ −1.94). Similarly, the high-income Asia-Pacific region also had the largest decrease in age-standardized DALY rates (EAPC -2.54, 95% CI: −2.68 ~ −2.39) (Table 1; Figures 1C,D). This overlap indicates that these countries face a particularly high burden of NAFLD, affecting both mortality and overall health-related quality of life.

### 3.4 Future projections of global NAFLD incidence burden

**Figure 2:**
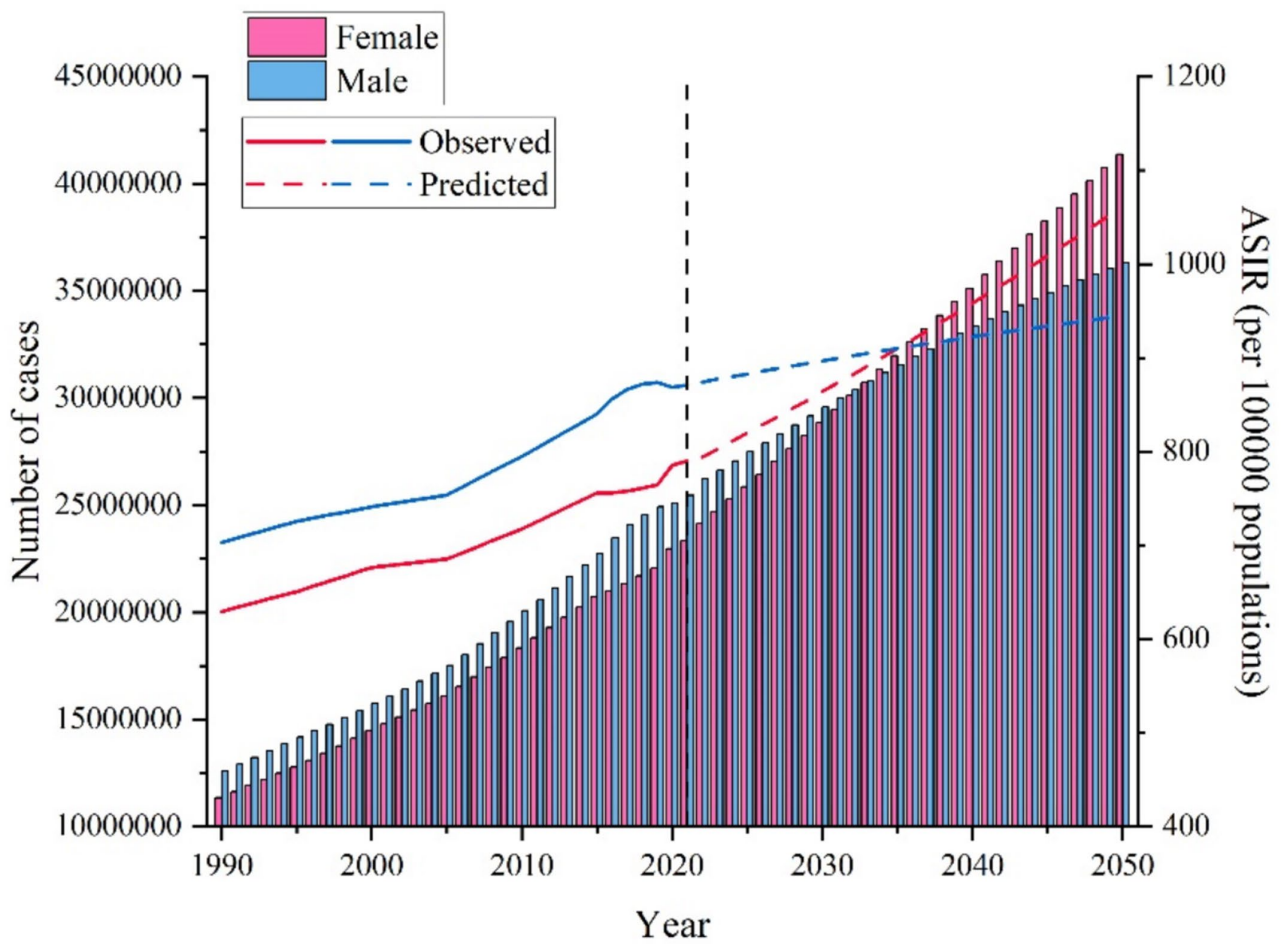
Future projections of global NAFLD incidence burden.

From 2021 to 2050, the global ASIR for NAFLD is expected to increase for both males and females. The male ASIR is projected to remain relatively stable, increasing slightly from approximately 820 per 100,000 in 2021 to 950 per 100,000 in 2050, representing a 15% increase over thirty years. In contrast, females are expected to experience a more significant increase, from approximately 800 per 100,000 in 2021 to 1,060 per 100,000 in 2050, representing a 32.5% increase over the same period, which is double the increase observed in males. Additionally, female ASIR is expected to surpass male ASIR after 2035 (see Figure 2).

### 3.5 Decomposition analysis of NAFLD epidemiological projections from 2020 to 2050

**Figure 3:**
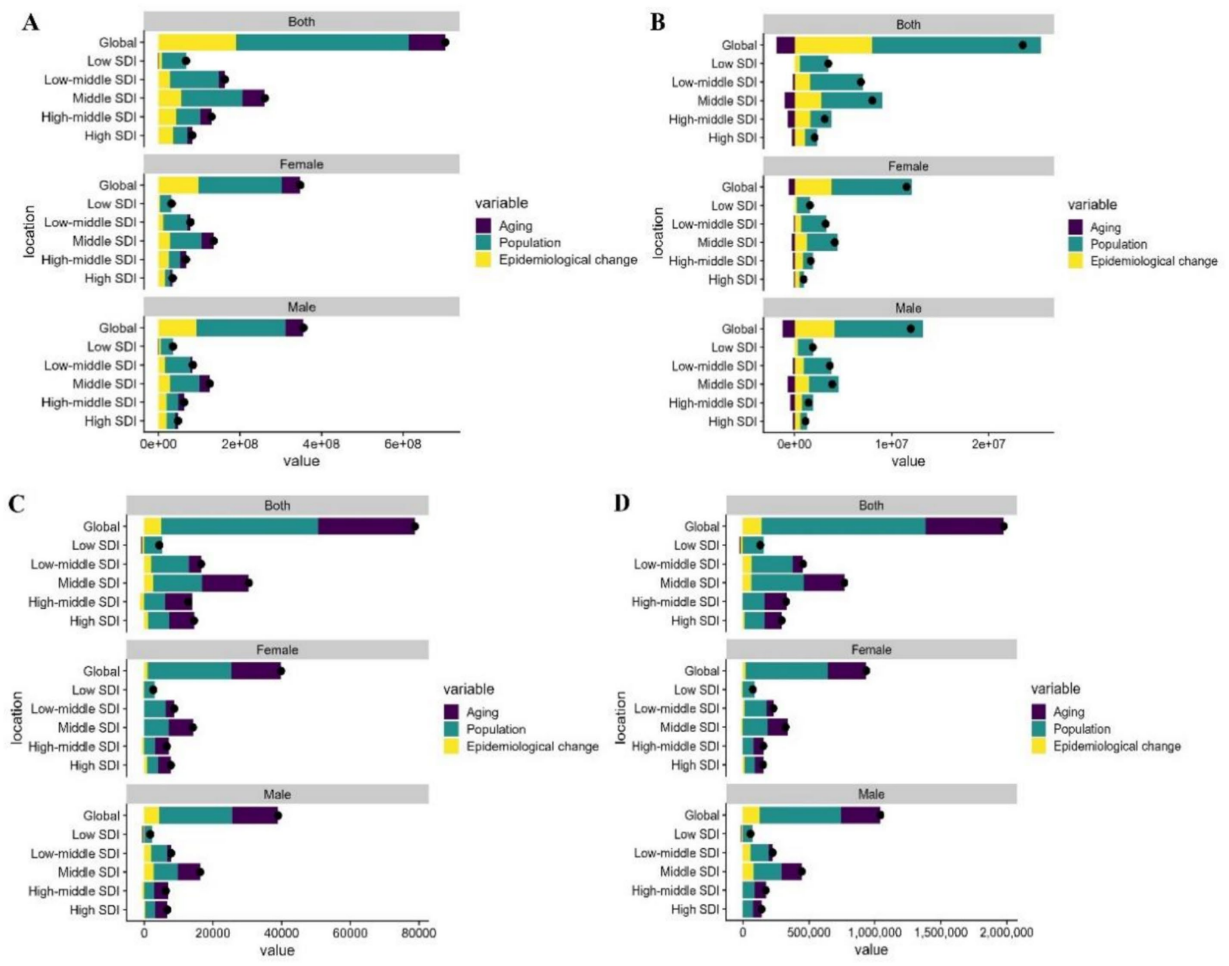
Decomposition Analysis of Global and Regional NAFLD Projections from 2020 to 2050. **(A)** Prevalence rate; **(B)** incidence rate; **(C)** death rate; **(D)** DALY rate.

The decomposition analysis shows the relative contributions of population aging, population growth, and epidemiological changes to the projected increase in NAFLD cases globally and by region. In the projected changes in prevalence, population growth is the primary factor, followed by population aging. Population growth has the greatest impact in low-middle SDI regions for both males and females, while population aging has the greatest impact in middle SDI regions (Figure 3A). Similarly, in the projected changes in incidence, population growth is also the primary factor, with the greatest impact in low-middle SDI regions. However, population aging has a negative contribution to the projected changes in incidence (Figure 3B). Population growth is the main driver of increases in mortality and DALY rates, followed by population aging. Changes in mortality and DALY rates have a minimal impact on projections (Figures 3C,D).

### 3.6 Frontier analysis of NAFLD mortality, DALY rates, and SDI from 1990 to 2021

**Figure 4:**
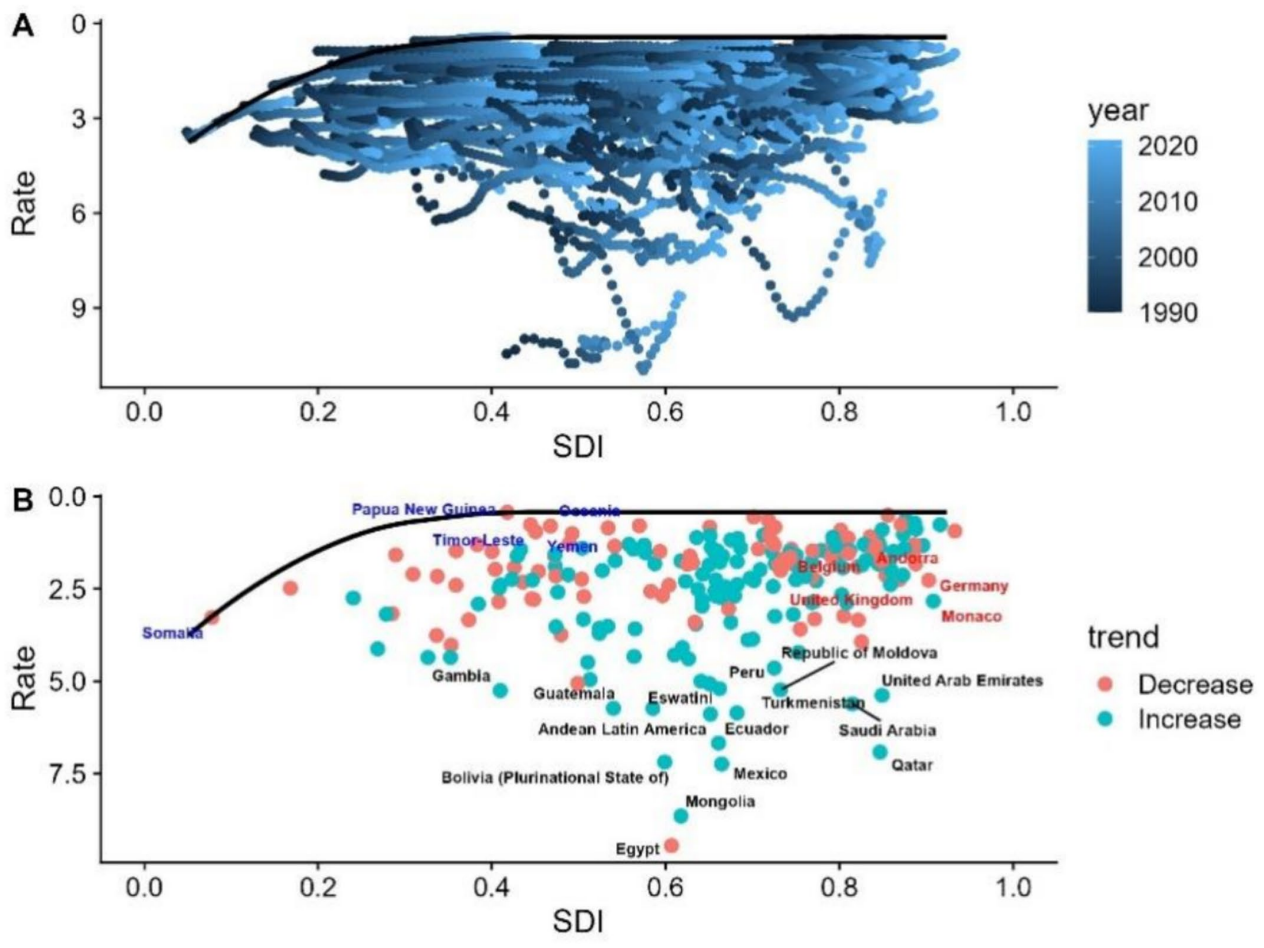
**(A)** Frontier analysis of NAFLD mortality and SDI from 1990 to 2021. **(B)** Frontier analysis of NAFLD mortality and SDI in 2021.

A frontier analysis was conducted based on NAFLD mortality and SDI data from 1990 to 2021 to better understand the potential for improving NAFLD mortality. The top 15 countries with the largest effective differences in the frontier include Gambia, Guatemala, Eswatini, Peru, Moldova, the United Arab Emirates, Latin America, Ecuador, Turkmenistan, Saudi Arabia, Bolivia, Mexico, Qatar, Mongolia, and Egypt. Compared to other countries, these countries have higher NAFLD mortality rates despite having comparable socio-demographic resources. Countries with low SDI (<0.5) and low effective differences in the frontier include Somalia, Papua New Guinea, Oceania, Timor-Leste, and Yemen. Belgium, Germany, Andorra, Monaco, and the United Nations are countries with high SDI (>0.85) and relatively high effective differences in development levels (Figures 4A,B).

**Figure 5:**
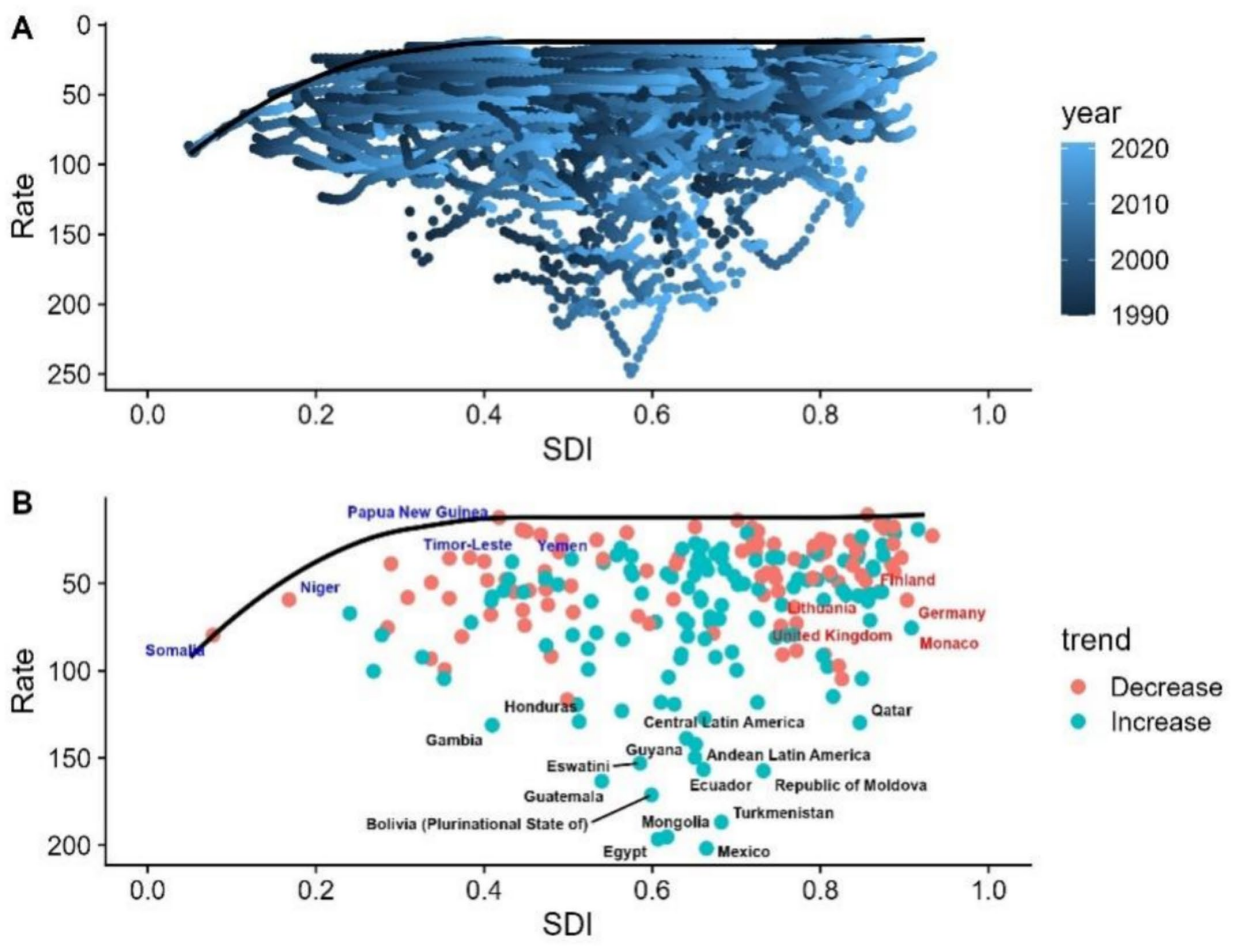
**(A)** Frontier analysis of NAFLD DALY rates and SDI from 1990 to 2021. **(B)** Frontier analysis of NAFLD DALY rates and SDI in 2021.

A frontier analysis was conducted based on NAFLD DALY rates and SDI data from 1990 to 2021 to better understand the potential for improving NAFLD DALY rates. The top 15 countries with the largest effective differences in the frontier include Gambia, Honduras, Central America, Guyana, Andean Latin America, Moldova, Eswatini, Ecuador, Guatemala, Turkmenistan, Bolivia, Mexico, Qatar, Mongolia, and Egypt. Compared to other countries, these nations have higher NAFLD DALY rates despite having comparable socio-demographic resources. Countries with low SDI (<0.5) and low effective differences in the frontier include Somalia, Niger, Papua New Guinea, Timor-Leste, and Yemen. Finland, Germany, the United Kingdom, Monaco, and the Netherlands are countries with high SDI (>0.85) and relatively high effective differences in development levels (Figures 5A,B).

## 4 Discussion

As one of the most prevalent chronic liver diseases globally, the trends and influencing factors of NAFLD burden are crucial for the formulation of public health policies. This study comprehensively assessed the global, regional, and national burden of NAFLD from 1990 to 2021 and conducted predictive analyses of future trends, providing important insights for understanding the epidemic dynamics of NAFLD and developing prevention and control strategies.

Globally, the prevalence, incidence, mortality, and DALY rates of NAFLD have exhibited significant upward trends. This study reveals that the global number of NAFLD cases in 2021 increased by 124.63% compared to 1990, with the ASPR and ASIR increasing at an annual rate of 0.73%. This trend is closely associated with the global epidemics of obesity, type 2 diabetes, and metabolic syndrome (6). For instance, environmental factors such as high-calorie diets and sedentary lifestyles directly exacerbate hepatic fat accumulation by promoting insulin resistance and lipid metabolism disorders (2). Additionally, the global number of NAFLD-related deaths in 2021 increased by 132.3% compared to 1990, with the ASDR increasing at an annual rate of 0.19%. This is primarily because approximately 20–30% of NAFLD patients may progress to non-alcoholic steatohepatitis (NASH) and advanced fibrosis (11), leading to increased liver-related mortality. Despite the strong association between NAFLD and metabolic syndrome, awareness of the disease and its prevention and treatment strategies have not been adequately integrated into global non-communicable disease control systems, which may significantly contribute to the continued rise in its burden (5).

At the regional level, the burden of NAFLD exhibits significant disparities. In 2021, the middle SDI region had the highest ASPR and ASIR, while the high SDI region had the lowest. This phenomenon may reflect more drastic lifestyle changes, such as Westernized diets and reduced physical activity, in middle SDI countries undergoing rapid urbanization (12). In contrast, high SDI regions benefit from more comprehensive disease screening and early intervention measures. The study also found that the high-middle SDI region experienced the largest increase in NAFLD burden. This may be related to economic development, accelerated urbanization, and the spread of unhealthy lifestyles in these regions. In comparison, the low SDI region had the smallest increase in ASPR, likely due to underdiagnosis or data collection biases rather than lower actual disease risk (13). These findings highlight the complex relationship between socio-economic development and NAFLD control capabilities, necessitating strategies to balance disease screening and healthcare investment.

At the national level, Oceania, Central America, and Latin America have the highest NAFLD prevalence, while North Africa and the Middle East have the highest incidence rates. The burden of NAFLD in these regions is closely tied to local dietary habits, obesity rates, and the prevalence of metabolic syndrome. Notably, Eastern Europe experienced the most significant increase in NAFLD mortality, indicating potential shortcomings in healthcare resource allocation and chronic disease management in these areas (8). Conversely, the high-income Asia-Pacific region saw the most pronounced decline in mortality, likely due to widespread obesity management programs (10). These differences may be attributed to variations in healthcare resources, public health policies, and awareness and control measures for NAFLD across regions.

Based on decomposition analysis, the projected increase in global NAFLD cases from 2021 to 2050 is primarily driven by population growth and aging. Female ASIR is expected to increase by 32.5%, significantly higher than that of males, potentially due to postmenopausal estrogen decline leading to lipid metabolism abnormalities (14). Additionally, low-middle SDI regions are most affected by population growth, while middle SDI regions are more impacted by aging. This suggests the need for differentiated intervention strategies in different regions. For example, lifestyle interventions should be promoted in regions with younger populations, while secondary prevention of metabolic complications should be strengthened in aging regions (15).

Although this study provides the first global predictive data on NAFLD from 1990 to 2021, it has limitations. First, GBD data rely on model estimates, which may underestimate the true burden in regions with underdiagnosis. Second, future projections do not account for the potential impact of emerging treatments or policy interventions. Future research should incorporate real-world data to validate model accuracy and explore optimal prevention and control strategies.

This study provides a comprehensive assessment of the global burden of NAFLD and offers valuable evidence for future prevention and control strategies. Further research should explore the pathogenesis of NAFLD and its association with metabolic syndrome to develop more effective prevention and treatment strategies.

## Data Availability

Publicly available datasets were analyzed in this study. This data can be found here: https://www.healthdata.org/gbd.
