# Supplementary material for: 1990–2021 global, regional, and national analysis of the burden and trends of non-alcoholic fatty liver disease
Source: Front Med (Lausanne). 2025 Jun 6;12:1609816. doi: 10.3389/fmed.2025.1609816 (PMC12179171; doi:10.3389/fmed.2025.1609816)
Supplement: Supplementary file 1 [file Table_1.docx]

**Appendix Table 1** Trends in the Disease Burden of NAFLD by Region from 1990 to 2021: Prevalence, Incidence, Mortality, and DALY Rate

| **Location** | **1990** | | **2021** | | **EAPC_95%CI** |
| --- | --- | --- | --- | --- | --- |
|  | **Number**  **（95%UI）** | **ASR（95%UI）** | **Number**  **（95%UI）** | **ASR（95%UI）** |  |
| **Prevalence** |  |  |  |  |  |
| Australasia | 1656063.54  (1512941.33~1804379.88) | 7376.03  (6738.42~8024.72) | 3778618.72  (3457855.40~4124388.06) | 9468.25  (8665.51~10349.36) | 0.83（0.80~0.87） |
| Andean Latin America | 3550474.82  (3245583.10~3891049.04) | 12946.17  (11857.20~14128.82) | 9737655.42  (8884037.84~10649706.29) | 14984.76  (13708.36~16361.47) | 0.53（0.51~0.55） |
| Caribbean | 4233435.21  (3871405.66~4631107.55) | 14073.50  (12862.56~15354.69) | 8111960.19  (7440860.48~8804729.26) | 15650.72  (14340.15~16986.20) | 0.38（0.37~0.40） |
| Central Asia | 7937267.64  (7270977.60~8700387.01) | 14118.98  (12962.74~15421.66) | 15204171.38  (13885517.00~16673758.37) | 16120.05  (14735.29~17604.48) | 0.45（0.41~0.48） |
| Central Europe | 15977945.95  (14560920.16~17364344.77) | 11366.94  (10414.56~12359.43) | 20606023.1  (18822095.87~22372105.07) | 12731.47  (11618.63~13852.64) | 0.39（0.37~0.41） |
| Central Latin America | 17471418.21  (16058559.55~19196192.83) | 14960.34  (13710.10~16309.99) | 44693566.17  (40900170.87~48782244.46) | 16983.98  (15536.52~18533.59) | 0.44（0.43~0.45） |
| Central Sub-Saharan Africa | 3761673.30  (3403642.87~4187887.80) | 10904.86  (9951.54~12009.31) | 10850617.54  (9833002.77~11986282.97) | 11870.63  (10844.86~12943.36) | 0.28（0.25~0.32） |
| East Asia | 142094264.50  (129881122.87~156713752.86) | 11080.88  (10133.33~12074.69) | 34696289.98  (31695847.38~37763370.68) | 15596.18  (14262.43~16999.34) | 0.67（0.46~0.89） |
| Eastern Europe | 28935071.09(26460106.18~31524213.95) | 12789.93(11676.75~14020.26) | 301408386.30(274406341.83~328824039.46) | 12293.91(11254.47~13359.17) | 0.32（0.31~0.33） |
| Eastern Sub-Saharan Africa | 13521292.58  (12352292.21~14926689.92) | 11774.95  (10738.60~12873.14) | 37304081.07  (34041055.12~41466665.55) | 13162.12  (12037.13~14400.23) | 0.35（0.33~0.37） |
| High-income Asia Pacific | 15202608.48  (13914421.41~16576612.98) | 7690.99  (7025.96~8379.61) | 24694242.29  (22636601.63~26784254.44) | 8885.73  (8148.40~9666.66) | 0.58（0.51~0.65） |
| High-income North America | 25568845.91  (23386397.91~27948788.71) | 7946.82  (7253.21~8661.46) | 48995594.51  (44673054.19~53423289.72) | 15182.69  (13936.16~16584.71) | 0.89（0.85~0.93） |
| North Africa and Middle East | 52713319.85  (48290123.13~57887147.52) | 21902.49  (20094.34~23849.75) | 164312588.80  (151441884.90~179050647.81) | 10056.02  (9187.35~10925.56) | 0.82（0.77~0.87） |
| Oceania | 605232.77  (551737.61~670031.92) | 13706.50  (12511.17~15026.99) | 1625692.90  (1483816.56~1796524.92) | 27686.69  (25586.92~29914.62) | 0.35（0.32~0.39） |
| South Asia | 99245062.09  (90560954.01~109619913.49) | 13984.92  (12785.15~15266.89) | 249790702.12  (227865116.89~273237523.38) | 14158.33  (12940.87~15445.08) | 0.43（0.35~0.51） |
| Southeast Asia | 49379730.32  (45181584.52~54280548.24) | 7995.98  (7321.85~8783.11) | 115103787.94  (104841847.81~125940612.95) | 15691.67  (14308.28~17127.24) | 0.39（0.38~0.40） |
| Southern Latin America | 3801551.25  (3476773.32~4182345.24) | 13932.66  (12701.43~15196.92) | 8080744.77  (7374983.85~8823030.23) | 10292.48  (9394.62~11265.42) | 0.84（0.80~0.88） |
| Southern Sub-Saharan Africa | 5251568.55  (4811688.05~5773195.25) | 12361.12  (11282.78~13532.19) | 11781789.25  (10763174.47~12907445.84) | 15937.24  (14572.60~17388.03) | 0.43（0.41~0.46） |
| Tropical Latin America | 18156836.23  (16647043.93~20103251.32) | 15001.90  (13772.66~16413.10) | 42870510.54  (39161072.13~46852272.07) | 16662.75  (15244.95~18205.46) | 0.39（0.37~0.40） |
| Western Europe | 38629112.96  (35378095.52~41988283.50) | 8144.98  (7490.53~8873.81) | 66258938.96  (60940043.36~71561630.34) | 10841.79  (9939.03~11801.96) | 0.98（0.93~1.02） |
| Western Sub-Saharan Africa | 16739352.57  (15345717.83~18454589.55) | 13317.94  (12164.76~14554.97) | 47962035.58  (43856186.89~53002936.55) | 14936.85  (13659.28~16347.60) | 0.35（0.33~0.36） |
| **Incidence** |  |  |  |  |  |
| Australasia | 62855.49  (56928.69~68897.80) | 288.08  (261.95~315.06) | 117958.49  (108097.06~127026.22) | 358.84  (328.05~390.77) | 0.72（0.68~0.76） |
| Andean Latin America | 171832.31  (155690.71~189720.27) | 494.93  (453.46~540.75) | 399223.32  (366915.68~433221.91) | 582.61  (536.86~630.67) | 0.55（0.54~0.56） |
| Caribbean | 178950.12  (162837.72~196254.67) | 513.74  (471.20~559.15) | 287884.02  (264113.47~310034.30) | 576.80  (528.70~623.41) | 0.42（0.41~0.44） |
| Central Asia | 347731.08  (317651.13~383978.01) | 530.14  (485.07~576.45) | 583558.98  (530899.64~636274.84) | 612.97  (560.97~665.27) | 0.52（0.47~0.56） |
| Central Europe | 548337.72  (502232.09~593266.01) | 419.01  (384.12~454.71) | 569426.06  (525602.35~613240.43) | 471.53  (433.07~510.90) | 0.40（0.38~0.43） |
| Central Latin America | 881456.39  (800893.37~971674.17) | 581.20  (531.70~632.78) | 1786199.46  (1641450.57~1939007.98) | 669.44  (615.97~726.19) | 0.48（0.47~0.49） |
| Central Sub-Saharan Africa | 193050.39  (173259.39~215527.73) | 434.61  (395.27~476.05) | 549882.74  (493162.38~610867.30) | 469.08  (426.91~513.72) | 0.22（0.19~0.26） |
| East Asia | 6397502.75  (5758117.66~7055942.25) | 495.87  (449.65~540.96) | 9905422.95  (9094622.58~10735336.06) | 620.37  (565.23~675.90) | 0.73（0.57~0.90） |
| Eastern Europe | 1003375.74  (918186.67~1086542.81) | 418.62  (384.11~454.77) | 1035049.77  (952601.26~1117151.81) | 471.81  (433.23~511.73) | 0.41（0.37~0.44） |
| Eastern Sub-Saharan Africa | 702062.14(625656.65~783545.72) | 468.64(425.87~512.55) | 1953106.13(1744597.89~2184436.96) | 522.51(475.30~570.84) | 0.36（0.34~0.38） |
| High-income Asia Pacific | 591333.67(539681.51~643475.20) | 310.56(282.75~338.69) | 728246.24(664400.95~786976.74) | 348.79(319.19~378.61) | 0.53（0.45~0.60） |
| High-income North America | 974201.22(886653.74~1063602.53) | 321.77(293.45~350.95) | 1635645.93(1495034.23~1770494.14) | 391.24(357.04~425.57) | 0.70（0.68~0.73） |
| North Africa and Middle East | 2621742.99(2382526.78~2885697.74) | 849.02(777.99~920.82) | 6578945.51(6067957.17~7080113.30) | 1037.64(963.01~1109.65) | 0.70（0.67~0.74） |
| Oceania | 31401.52(28265.90~34937.84) | 542.51(493.89~593.47) | 76653.58(69527.75~84514.60) | 588.57(538.68~640.66) | 0.27（0.23~0.30） |
| South Asia | 4362687.94(3921919.59~4818166.71) | 464.89(421.18~507.16) | 10765350.57(9758063.81~11817448.59) | 564.19(513.27~615.45) | 0.61（0.55~0.67） |
| Southeast Asia | 2347042.81(2125152.57~2601001.33) | 541.66(491.92~590.09) | 4606197.41(4201349.07~5033587.85) | 622.70(569.31~678.69) | 0.48（0.47~0.50） |
| Southern Latin America | 154123.81(140379.97~168851.10) | 313.83(285.65~343.42) | 283022.26(258970.01~307037.96) | 386.42(352.60~420.05) | 0.68（0.65~0.72） |
| Southern Sub-Saharan Africa | 273213.00(245837.16~304043.01) | 570.40(520.52~622.36) | 532714.01(484100.80~584242.02) | 651.93(597.18~709.27) | 0.47（0.46~0.48） |
| Tropical Latin America | 806667.91(732235.24~888591.41) | 557.12(509.05~604.17) | 1584117.69(1456856.30~1706172.50) | 648.83(596.36~700.52) | 0.55（0.53~0.57） |
| Western Europe | 1387072.89(1270984.83~1508210.43) | 324.92(296.52~354.41) | 1871635.08(1719781.98~2008903.80) | 401.69(368.55~435.70) | 0.73（0.69~0.78） |
| Western Sub-Saharan Africa | 818838.77(737068.54~907475.42) | 521.28(474.89~569.13) | 2501855.88(2243383.61~2767096.32) | 597.04(544.27~651.74) | 0.44（0.43~0.45） |
| **Deaths** |  |  |  |  |  |
| Australasia | 180.28(136.71~232.16) | 0.78(0.60~1.01) | 655.72(522.32~805.17) | 1.27(1.01~1.55) | 1.77（1.64~1.91） |
| Andean Latin America | 973.92(676.24~1326.40) | 4.73(3.32~6.45) | 3442.52(2362.27~4733.75) | 5.89(4.03~8.07) | 0.71（0.63~0.79） |
| Caribbean | 724.41(521.23~959.47) | 2.82(2.04~3.72) | 1601.54(1134.66~2196.48) | 2.97(2.11~4.07) | 0.13（0.12~0.37） |
| Central Asia | 1010.95(752.43~1334.27) | 2.17(1.60~2.86) | 2754.17(1993.36~3732.16) | 3.41(2.47~4.59) | 1.66（1.43~1.88） |
| Central Europe | 2018.81(1503.83~2694.29) | 1.37(1.02~1.79) | 3502.74(2567.91~4721.40) | 1.69(1.24~2.27) | 0.35（0.20~0.50） |
| Central Latin America | 3586.23(2590.48~4760.52) | 4.21(3.04~5.56) | 12632.45(9451.92~16291.33) | 5.00(3.74~6.45) | 0.56（0.48~0.63） |
| Central Sub-Saharan Africa | 396.04(261.84~625.09) | 1.76(1.14~2.85) | 908.03(592.21~1384.89) | 1.61(1.04~2.50) | -0.41（  -0.49~-0.33） |
| East Asia | 8691.10(6737.43~10950.25) | 1.04(0.80~1.32) | 17603.54(13572.65~21977.57) | 0.83(0.65~1.04) | -0.54（-0.65~-0.42） |
| Eastern Europe | 2548.03(1873.55~3418.75) | 0.92(0.68~1.22) | 8415.16(6162.07~11402.40) | 2.66(1.93~3.62) | 3.58（2.95~4.22） |
| Eastern Sub-Saharan Africa | 1589.04(1185.58~2108.52) | 2.28(1.68~3.04) | 3812.03(2856.60~5042.54) | 2.40(1.79~3.19) | 0.04（-0.02~0.09） |
| High-income Asia Pacific | 3100.53(2443.53~3820.10) | 1.57(1.24~1.94) | 4549.56(3414.88~5705.61) | 0.87(0.67~1.08) | -2.09（-2.24~-1.94） |
| High-income North America | 3670.06(2801.02~4717.99) | 1.07(0.82~1.40) | 9817.14(7582.68~12378.89) | 1.56(1.21~1.97) | 1.44（1.30~1.59） |
| North Africa and Middle East | 3676.27(2497.01~5486.92) | 2.64(1.74~4.06) | 11003.53(8016.00~14951.57) | 2.70(1.93~3.68) | 0.10（0.02~0.18） |
| Oceania | 27.14(17.77~44.87) | 0.89(0.58~1.48) | 61.79(43.99~87.03) | 0.81(0.57~1.13) | -0.52（-0.64~-0.41） |
| South Asia | 6427.59(4554.21~9106.78) | 1.10(0.77~1.57) | 18670.34(13608.38~24920.86) | 1.30(0.93~1.73) | 0.51（0.45~0.56） |
| Southeast Asia | 4005.08(2887.32~5741.19) | 1.62(1.15~2.40) | 11497.81(8410.11~15006.30) | 1.86(1.35~2.40) | 0.46（0.40~0.53） |
| Southern Latin America | 500.41(360.36~708.79) | 1.79(1.24~2.44) | 1475.65(1068.81~1985.11) | 1.70(1.23~2.29) | 0.26（0.14~0.38） |
| Southern Sub-Saharan Africa | 820.33(566.92~1122.61) | 1.84(1.29~1.55) | 1575.32(1246.46~1975.64) | 2.80(2.21~3.48) | 1.16（0.72~1.61） |
| Tropical Latin America | 1126.24(840.07~1494.11) | 1.18(0.89~1.55) | 3568.91(2678.06~4594.11) | 1.38(1.04~1.77) | 0.76（0.66~0.86） |
| Western Europe | 12183.10(8870.78~15745.80) | 2.17(1.60~2.80) | 15693.80(11844.45~19572.36) | 2.77(2.11~3.62) | -0.75（-0.90~-0.59） |
| Western Sub-Saharan Africa | 2280.87(1547.13~3454.45) | 2.77(1.85~4.24) | 5086.51(3836.64~6732.15) | 1.75(1.34~2.20) | -0.05（-0.08~-0.02） |
| **DALY** |  |  |  |  |  |
| Australasia | 4964.67（3775.34~6480.57） | 21.93（16.69~28.86） | 15506.5（12409.52~18959.16） | 33.24（26.63~40.62） | 1.63（1.49~1.77） |
| Andean Latin America | 28440.96（19672.01~38623.48） | 124.44（86.26~170.3） | 86181.43（60088.12~120245.2） | 142.16（98.6~198.78） | 0.35（0.25~0.45） |
| Caribbean | 20067.57（14402.3~26824.5） | 73.84（52.88~98.81） | 42917.07（30008.94~59389.84） | 80.31（56.31~110.84） | 0.25（0.00~0.51） |
| Central Asia | 29161.99（21978.01~38323.64） | 57.94（43.86~76.18） | 83069.77（60166.12~114191.04） | 92.24（67.18~124.97） | 1.59（1.34~1.84） |
| Central Europe | 56464.75（41615.24~75849.7） | 38.08（28.09~51.07） | 90826.25（66040.27~123717.82） | 47.9（34.85~64.84） | 0.33（0.15~0.51） |
| Central Latin America | 110821.95（79839.53~148645.05） | 115.68（82.48~156.37） | 362135.88（270505.51~471168.84） | 138.84（104.37~180.55） | 0.54（0.45~0.64） |
| Central Sub-aharan Africa | 13061.98（8514.08~20012.74） | 47.73（31.27~74.29） | 30509.83（19961.11~45913.37） | 43.12（28.16~65.78） | -0.44（-0.52~-0.35） |
| East Asia | 263442.06（202680.21~330422.56） | 27.43（21.28~34.39） | 435186.46（336685.89~552061.74） | 20.11（15.61~25.11） | -0.92（-1.04~-0.80） |
| Eastern Europe | 72748.18（53015.1~98819.97） | 26.28（19.11~35.14） | 270210.57（193015.92~372709.72） | 91.4（65.18~125.26） | 4.09（3.28~4.90） |
| Eastern Sub-Saharan Africa | 47076.44（35632.39~61641.93） | 55.99（41.95~74.00） | 113483.55（85887.52~151390.99） | 57.52（42.96~76.31） | -0.06（-0.12~0.00） |
| High-income Asia Pacific | 77856.3（63124.31~97106.45） | 37.92（30.69~47.13） | 79466.06（61381.43~97546.38） | 18.75（14.82~23.05） | -2.54（-2.68~-2.39） |
| High-income North America | 98110.34（74311.55~129086.9） | 30.13（22.32~39.78） | 241654.94（188286.87~307000.45） | 42.07（32.96~54.17） | 1.36（1.21~1.51） |
| North Africa and Middle East | 90349.36（63408.89~124268.31） | 53.96（37.45~78.19） | 276311.16（205334.42~374125.68） | 58.96（43.63~79.78） | 0.36（0.32~0.40） |
| Oceania | 937.18（603.84~1487.96） | 25.02（16.37~40.77） | 2058.09（1462.55~2894.34） | 22.07（15.71~31.07） | -0.60（-0.72~-0.48） |
| South Asia | 209501.36（151595.72~294195.85） | 30.47（21.52~43.06） | 540637.88（397352~722267.03） | 33.74（24.71~44.57） | 0.28（0.25~0.31） |
| Southeast Asia | 120546.2（87922.2~169965.71） | 41.58（30.11~59.29） | 313318.09（235052.09~411932.25） | 45.34（34.02~58.79） | 0.27（0.21~0.34） |
| Southern Latin America | 23155.37（16241.29~32045.25） | 49.62（34.83~68.63） | 36865.14（26789.26~50789.93） | 43.99（31.93~60.55） | 0.08（-0.05~0.21） |
| Southern Sub-Saharan Africa | 15806.25（11727.39~21316.82） | 49.55（36.00~69.25） | 47069.22（37281.53~59698.81） | 72.65（57.59~91.06） | 1.08（0.60~1.56） |
| Tropical Latin America | 37574.9（27874.57~50072.57） | 34.59（25.32~46.35） | 101282.57（74624.99~133351.26） | 38.55（28.63~50.46） | 0.53（0.42~0.63） |
| Western Europe | 304934.16（225758.34~399476.41） | 58.35（42.69~76.44） | 349703.39（269836.29~441853.17） | 45.31（35.03~57.09） | -0.89（-1.07~-0.71） |
| Western Sub-Saharan Africa | 64232.13（44112.59~93542.84） | 66.57（45.48~98.09） | 148873.3（110156.35~198127.71） | 65.31（49.29~86.28） | -0.11（-0.15~-0.07） |
